# A Case of New-Onset Atrial Fibrillation With Rapid Ventricular Response Due to Iatrogenic Hypothermia

**DOI:** 10.7759/cureus.23822

**Published:** 2022-04-04

**Authors:** Hany A Zaki, Mubarak Alhatemi, Mohamed Hendy, Yasser Kaber, Haris Iftikhar

**Affiliations:** 1 Emergency Medicine, Hamad Medical Corporation, Doha, QAT

**Keywords:** postoperative deterioration, iatrogenic complication, cardiac arrythmia, hypothermia, atrial fibrillation (af)

## Abstract

Hypothermia is an involuntary fall in body temperature, usually below 35°C. Hypothermia is a common condition, especially in frigid zones. However, it should not be forgotten that it can also occur in temperate climates or for iatrogenic reasons. Hypothermia is associated with seriously severe arrhythmias, particularly ventricular fibrillation, and there are many reports of prolonged resuscitation in these patient groups. This case report shows that a standard thermometer, either with Emergency Medical Services or in-hospital, will be incapable of reading the temperature if it is less than 34°C and will falsely read 34°C when in reality it is lower than that; in a clinically relevant scenario, a low-reading thermometer or core body temperature readings, such as rectal or esophageal, should be used.

## Introduction

Atrial fibrillation (AF) is a high prevalence form of supraventricular tachycardia. It is a common condition in current clinical settings [1]. In the last two decades, hospital admissions for atrial fibrillation have increased by over 66%. It is expected to increase further due to the aging population, increasing prevalence of chronic heart disease, and advances in diagnostic and monitoring devices [2-7]. Atrial fibrillation is one of the most costly conditions treated in hospitals in the United States today [5]. Hospitalization accounts for a large portion of this amount, followed by consultations, job losses, and health care expenditures [4].

Hypothermia is defined as an involuntary drop in body temperature, usually below 35°C. Hypothermia is a common condition, especially in cold regions. However, it should not be forgotten that it can also occur in temperate climates or for iatrogenic reasons [8]. Symptoms of hypothermia vary in severity. The severity of this condition is defined as mild, moderate, or severe (relative to core temperature). Mild hypothermia is classified as 32°C to 35°C, moderate as 28°C to 32°C, and severe below 28°C. Some authorities have categorized certain patients with profound hypothermia (< 24°C). More severe symptoms, including morbidity and mortality, are associated with extremely low degrees of hypothermia [9]. Hypothermia is associated with severe cardiac arrhythmias, particularly ventricular fibrillation, and there are many reports of prolonged resuscitation in these patient groups [10-14].

In this case report, we present a patient with new-onset atrial fibrillation with a rapid ventricular response after iatrogenic hypothermia.

## Case presentation

**Figure 1:**
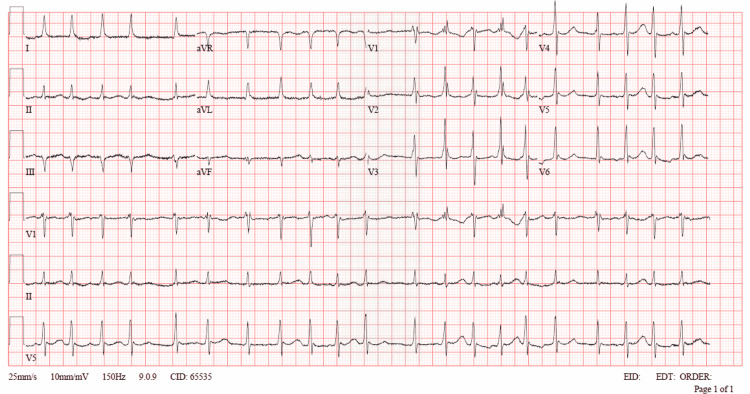
ECG showed Atrial fibrillation with the rapid ventricular response with aberrant conduction, Non-specific ST - T abnormality, with possible right ventricular conduction delay (RSR "QR" in V1 and V2).

A 51-year-old female patient with a history of diabetes mellitus and migraine underwent liposuction and bilateral brachioplasty. She was brought to the emergency department due to the development of blurred consciousness after the operation. The patient, who was extubated without any problem after the operation, was then re-intubated due to the development of blurred consciousness. When the patient came, her pulse was 133/min, respiratory rate was 16/min, blood pressure was 110/70 mmHg, oxygen saturation (SpO2) was 100%, and body temperature was 34°C. Her core temperature with a rectal probe was 30.5°C. In the physical examination, the patient was on a mechanical ventilator, bilateral equal air inflow was present and no additional sound was heard. She was tachycardic and rhythm was irregular, and her ECG showed atrial fibrillation with a rapid ventricular response (Figure 1).

Bedside ultrasound showed good cardiac contractility. There was no pericardial effusion or right ventricular dilatation. No free fluid was observed in the abdomen. The patient's arterial blood gas showed metabolic acidosis. Ketone body was negative, and the lactate level was 5.30 mmol/L. All other laboratory values ​​were within normal limits.

**Figure 2:**
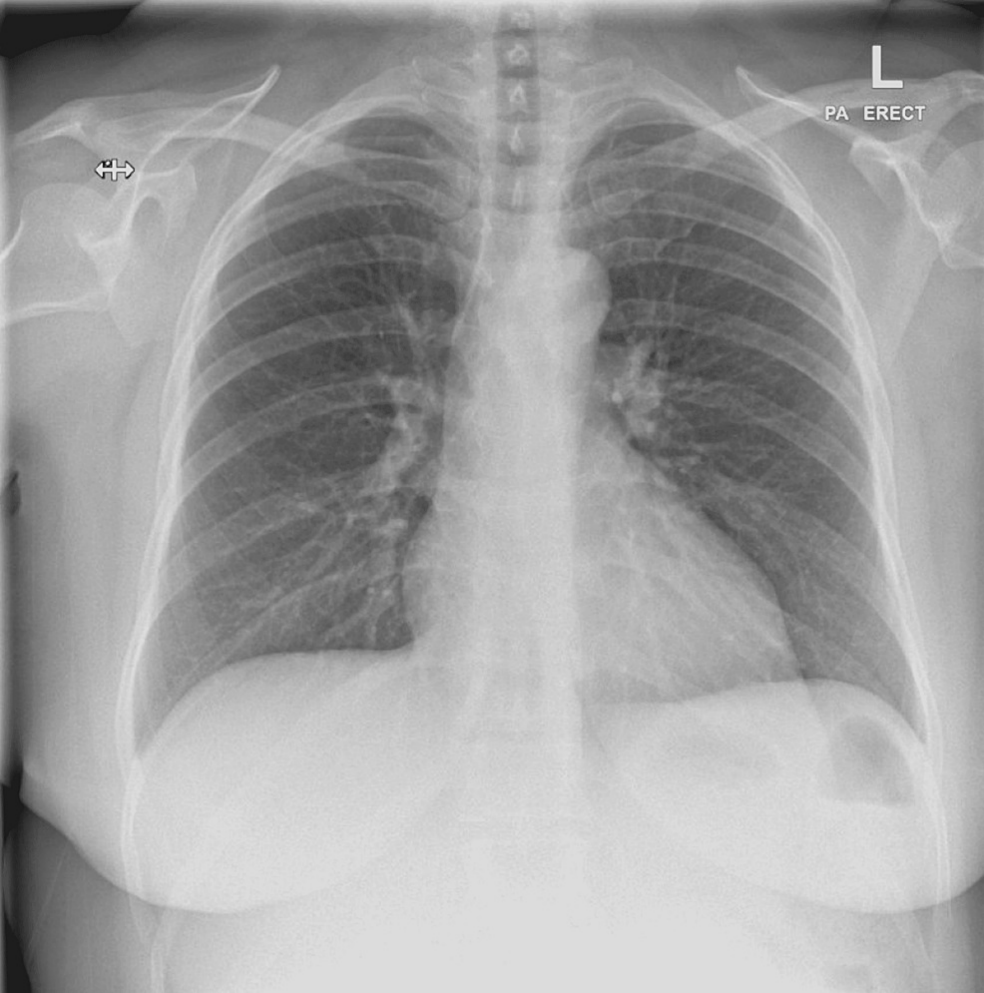
Chest x-ray showed no obvious patches or consolidations with normal costophrenic angles and cardiothoracic ratio.

Normal saline at 40°C was immediately given. The patient was tried to be warmed with reusable warming unit and single-use disposable warming blankets. The patient moved all her limbs, opened her eyes, and obeyed commands shortly after raising her core body temperature. We kept the patient under minimal sedation with propofol and fentanyl. The patient was admitted to the surgical intensive care unit for observation. After the core body temperature has risen, the rhythm spontaneously reverted to sinus rhythm. the patient was extubated chest x-ray was done and showed no patch or consolidation (Figure 2). The patient was then discharged completely asymptomatic. This case highlights that the ordinary thermometer with Emergency Medical Service or hospital cannot record temperature if below 34°C and will falsely say it is 34°C when in reality it is lower than that. When in doubt in a clinically relevant scenario, a low-reading thermometer or core body temperature readings like rectal thermometry or esophageal should be used.

## Discussion

Sinus tachycardia is a common condition in mild hypothermia. It usually occurs with the presence of a flickering artifact. ECG tremor artifact is commonly observed in patients with mild hypothermia. In some cases, clinicians may confuse it with atrial fibrillation, especially when there is underlying sinus arrhythmia. Atrial fibrillation is the most common tachyarrhythmia reported in people with severe hypothermia [15]. Graham et al. reported that the incidence of atrial fibrillation was 21% in prospective studies. This observation was consistent with an incidence of 19% previously reported by Vassallo et al. However, it is important to note that this was a small population study in both series. Artifacts that may occur due to hypothermia in hypothermic patients may cause difficulties in diagnosing atrial fibrillation [16-18]. Vassallo's study found that a regularly occurring rhythm represents a sinus mechanism, compared to a rhythm with an irregular appearance despite an uninterpretable baseline based on ECG artifacts from patients with similar rhythms [19]. Core body temperature at 86°F (30°C) caused a tremendous increase in myocardial irritability, resulting in ectopic beats that eventually progressed to atrial fibrillation [20,21]. There are some conflicting statements regarding its relationship with mortality. As Okada reported in 1984, there was no significant difference between the atrial fibrillation and non-atrial fibrillation groups [15]. In a prospective study by Rankin and Rae [22] in 1984, atrial fibrillation was reported as a benign rhythm in the atrial fibrillation group compared to the non-atrial fibrillation group.

The effects of hypothermia on the ECG of patients with atrial fibrillation in induced hypothermia were reported by Somerville in 1960. They reported that the atrial and ventricular rate slowed as the temperature dropped by 1 - 3°C. The rate dropped continuously until the temperature dropped to about 40 per minute. Further reductions changed the QRS morphology. In nine patients, the ventricular rate became regular after a 1-3°C drop in temperature, although atrial fibrillation still persisted. Rhythm returned to baseline after reversal of hypothermia. This was attributed to hypothermia with significant AV suppression with independent activity in connection with a pacer above the bifurcation of the His bundle. According to the study, atropine increased the rate and restored the irregular rhythm, suggesting that a chemically mediated mechanism may play a role outside of the direct effect of hypothermia [23].

There is no doubt that cardiac arrhythmia in hypothermia is well documented. It is important to note that atrial fibrillation is a common form of benign arrhythmia. It usually reverts to sinus rhythm during rewarming of the patient. Ventricular systoles may occur, but they are not precursors of ventricular tachycardia. Ventricular fibrillation occurs at low temperatures and is considered the leading cause of death in induced hypothermia [10,24].

## Conclusions

This case shows that an ordinary thermometer with Emergency Medical Services or hospital will not be able to record the temperature if it is below 34°C and will falsely say 34°C when in reality it is lower than that. In a clinically relevant scenario, readings should be taken with a low recording thermometer or a core body temperature recorder such as a rectal or esophageal thermometer.
